# Microbial metabolism in deep terrestrial subsurface communities - amino acids as biosignatures

**DOI:** 10.1016/j.crmicr.2026.100547

**Published:** 2026-01-03

**Authors:** Merja Herzig, Malin Bomberg, Tuulia Hyötyläinen

**Affiliations:** a Department of Nuclear Chemistry, Faculty of Nuclear Sciences and Physical Engineering, Czech Technical University in Prague, Czech Republic; b VTT Technical Research Centre of Finland, 02150 Espoo, Finland; c School of Science and Technology, EnForce, Environment and Health and Systems medicine, Örebro University, SE-701 82 Örebro, Sweden

**Keywords:** Subsurface, Bacterial metabolism, Bacterial metabolites, Microbial communities

## Abstract

The deep terrestrial subsurface (DTS) biosphere consists of a variety of distinct microbial taxa, mostly bacterial. The mechanisms by which microbes dynamically manage the uptake and concurrent utilization of nutrients within the DTS environments remain largely unexplored. Here, we examined the utilization patterns of amino acids and other polar metabolites in cultured DTS bacterial communities to investigate the adaptive responses and metabolic pathways employed under varying nutrient conditions to gain insight into how environmental shifts impact the metabolism of these communities. Previously, we found that changes in growth conditions affected the composition and size of the bacterial communities enriched from these oligotrophic, anoxic environments and induced changes in the production of primary and secondary metabolites. In the present study, metabolic fingerprinting was used to investigate the primary and secondary metabolite utilization and main metabolic pathways present in the enriched DTS bacterial consortium originating from the deep bedrock of the Fennoscandian Shield. We found that especially amino acids were predominantly degraded under different nutrient conditions. Notably, the degradation of phenylalanine and valine constituted a 'core' metabolic process that remained unaffected by variations in available nutrients within this community. Further, the most significant metabolic pathways employed were those connected to phenylalanine, cysteine and methionine.

## Highlights

• Salinity and nutrient availability affect the utilization and secretion of amino acids by deep biosphere microbial consortia.
• Anoxic deep biosphere enrichment cultures were compared to *Desulfovibrio desulfuricans*.
• Amino acids and organic acids were degraded under different nutrient conditions.
• Phe and Val degradation remained unaffected by changes in nutrient availability.
• Metabolic pathways involving Phe, Cys and Met were most prominent.

## Graphical abstract

**Figure:**
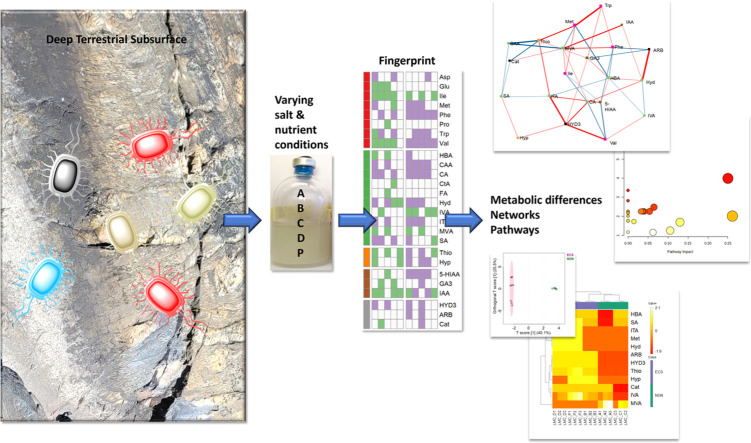

## 1 Introduction

In natural environments, bacteria face continuous fluctuations in nutrient availability, necessitating rapid metabolic adaptations to ensure their survival (Arnoldini et al., 2012; Schuetz et al., 2012). To achieve this, microbial cells process a variety of environmental signals to determine which nutrients to consume and when to switch to other available resources (Link et al., 2013). During these processes, complex biochemical reaction networks are formed (Zhang et al., 2020). Metabolic fingerprinting can be used to study the variations in these networks in response to environmental changes or different conditions, and to assess the state of the environmental system by examining the composition, connections, and alterations of the metabolite fingerprints (Coen et al., 2008; Zhang et al., 2020).

However, deciphering changes in metabolic networks is particularly challenging in complex media where multiple nutrients are simultaneously consumed and produced. New analytical methodologies that simultaneously measure changes in the abundance of multiple metabolites can deepen our understanding of deep terrestrial subsurface (DTS) (for list of abbreviations, see Table 1) metabolism and provide insights into how bacteria adapt to complex and dynamic nutritional conditions (Zampieri et al., 2019). Here, we used data from combined target-non-target mass-spectrometry of polar metabolites in the DTS enriched bacterial community (Herzig et al., 2024) to understand how bacteria adapt their metabolism in complex chemical environments with changing nutrient availability. There is considerable interest in understanding the metabolism of DTS microbial communities, particularly among industries such as CO_2_ capture and storage (CCS) and deep geological disposal of hazardous waste, which rely on the stability of the deep subsurface environment. However, despite the increased interest in the deep biosphere over the past decades, its microbiology, and especially microbial metabolism, remains poorly understood.

**Table 1:** Abbreviations used in the text.

*General abbreviations*		*Metabolite abbreviations*	
*ALL-MR*	*Low salinity solution*	*3HYD/HYD3*	*3-Hydroxy–4–5-dimethylfuran-2(5H)-one*
*BAS*	*Baseline media*	*5-HIAA*	*5-Hydroxyindole-3-acetic acid*
*CCS*	*Carbon dioxide Capture and Storage*	*ARB*	*Arbutin*
*DD*	*Desulfovibrio desulfuricans type strain 642*	*Asp*	*Aspartic acid*
*DSPC*	*Debiased Sparse Partial Correlation analysis*	*Cat*	*Catechin*
*DTS*	*Deep Terrestrial Subsurface*	*CAA*	*cis-dihomoaconitic acid*
*ECS*	*Exogenous organic Carbon Source*	*CtA*	*Citric acid*
*LMC*	*Bacterial mixture from deep bedrock site in Liminka, Finland*	*CA*	*Coumaric acid*
*NON*	*Cultivation without ECS (Exogenous organic Carbon Source)*	*FA*	*Fumaric acid*
*OL-SR*	*High salinity solution*	*GA3*	*Gibberelic acid*
*OPLS-DA*	*Orthogonal Partial Least Squares Discriminant Analysis*	*Glu*	*Glutamic acid*
*PG*	*Postgate medium*	*HBA*	*Hydroxybutyric acid*
*SRB*	*Sulfate reducing bacterium*	*HMF*	*5-(hydroxymethyl)furfural*
		*Hyd*	*Hydroxyglutaric acid*
***Bacteria abbreviations***		*Hyp*	*Hypoxanthine*
*Ace*	*Acetobacterium*	*IAA*	*Indole-3-acetic acid*
*Ars*	*Arsenicicoccus*	*Ile*	*Isoleucine*
*Clo*	*Clostridia*		
*C13*	*Clostridium sensu stricto 13*	*IVA*	
*Com*	*Comamonadaceae*	*ITA*	*Itaconic acid*
*Desm*	*Desulfomicrobium*	*Met*	*Methionine*
*Desv*	*Desulfovibrio*	*MVA*	*Mevalonic acid*
*Fir*	*Firmicutes*		
*Hyd*	*Hydrogenophaga*	*Phe*	*Phenylalanine*
*Lac*	*Lactobacillales*	*Pro*	*Proline*
*NA*	*bacteria with no further classification*	*SA*	*Succinic acid*
*Pol*	*Polaromonas*	*Thio*	*2-Thiocytidine dihydrate*
*Pro*	*Proteiniphilum*	*Trp*	*Tryptophan*
*Pse*	*Pseudomonas*	*Val*	*Valine*
*Rho*	*Rhodobacteraceae*		
*Sph*	*Sphingorhabdus*		
*Xan*	*Xanthobacter*		

The DTS environments host a diverse array of bacterial species competing for space and resources, with their abundance and composition being crucial to the overall metabolism detected in the DTS (van Tatenhove-Pel et al., 2021). Furthermore, the availability and concentration changes of exogenous organic carbon sources (ECS) significantly impact the population, community structure, and ecological function of DTS microorganisms (e.g. Herzig et al., 2024). Further, suitable substrates, like methanol and acetate readily activate the metabolism of dormant DTS microorganisms (Rajala et al., 2015; Rajala and Bomberg, 2017; Bomberg et al., 2017; Miettinen et al., 2018), and e.g. activation of dissimilatory sulfate reduction pathway has been suggested in enriched DTS bacterial communities after ECS introduction (Herzig et al., 2024).

Many nonequilibrium electron transfer reactions in the environment arise from microbial activity using ancient, conserved pathways. In deep subsurface environments, anaerobic microbial processes drive C, H, and S cycles, including methanogenesis, fermentative H₂ production, and organotrophic sulfate respiration, together with organic acids or hydrocarbon oxidation (Falkowski et al., 2008). Key bacteria include sulfate-reducing bacteria (SRB) such as *Desulfovibrio* and *Desulfomicrobium* (Xiong et al., 2016). However, while SRB play central roles in C and S cycling (Kwon et al., 2016), their influence on DTS community structure and total metabolism remains largely unknown and the research on the overall microbial metabolites and microbial metabolic pathways, including regulatory mechanisms, in DTS environments are to date unclear. Therefore, we hypothesize that change in environmental conditions, such as salinity and availability of ECS is strongly reflected in the secretion and utilization patterns of amino acids and other organic acids of DTS microbiomes. These patterns may strongly influence the conditions of deep geological storage and disposal sites. It is thus of great significance to identify these changes in microbial community structure and metabolic patterns responding to changes in environmental conditions in these habitats.

Previously we studied the primary and secondary metabolites produced by two deep bedrock subsurface bacterial enrichments cultures from the Fennoscandian Shield cultured under varying simulated groundwater conditions using high-throughput amplicon sequencing, 16S rRNA gene and *dsr*B gene targeting qPCR and ultra-high-performance liquid chromatography quadrupole time-of-flight mass spectrometry (UHPLC-QTOFMS) for metabolite determinations (Herzig et al., 2024). In the current study we analyzed metabolic pathways and degradation patterns in a DTS enrichment culture to construct a comprehensive metabolic fingerprint and compared it with the reference SRB strain *Desulfovibrio desulfuricans* (type strain 642) to identify differences between canonical SRB metabolism and community-level metabolism in DTS. Although DTS enrichment cultures do not fully replicate in situ communities, they offer a much closer approximation than using reference strains alone and, when combined with reference strains, enable assessment of shifts in metabolic structure across the broader community.

## 2 Materials and methods

### 2.1 Data

In this study, we used the previously obtained data from (Herzig et al., 2024 (https://doi.org/10.1111/1462-2920.16552), Supplement 3, and https://www.ebi.ac.uk/ena/, Study number PRJEB67711) for metabolic fingerprinting and pathway analysis.

**Fig. 1:**
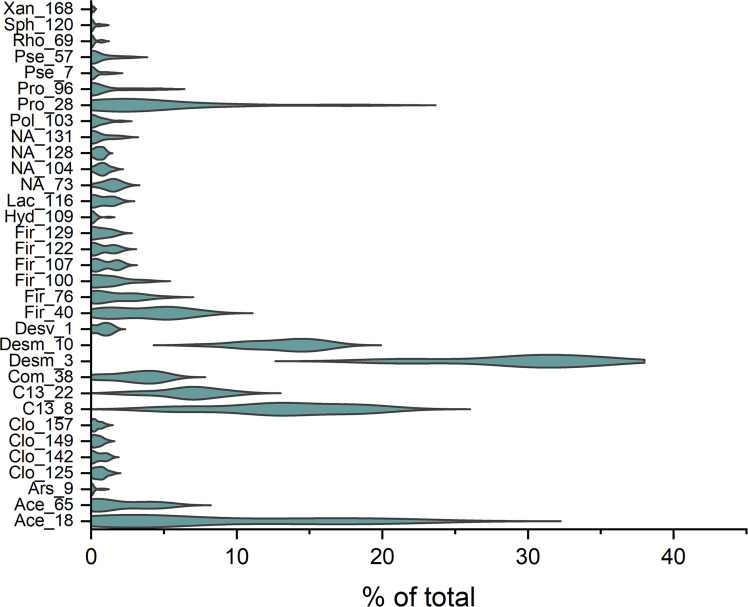
The major bacterial ASVs belonging to identified genera (ASVs > 10 sequence counts) in LMC (% of the bacterial consortia). For abbreviations, see Table 1. The numbers stand for corresponding ASVs (see https://www.ebi.ac.uk/ena/, Study number PRJEB67711) (Data from Herzig et al., 2024).

Data originated from a bacterial mixture from a deep bedrock site in Finland (Liminka, 600 m (LMC)) (Herzig et al., 2024). Additionally, *Desulfovibrio desulfuricans* type strain 642 (DD) was included as a reference SRB due to its central role in anoxic DTS environments and suitability for evaluating community metabolism (Herzig et al., 2024). The LMC sample was collected and cultivated as described in Noroaho (2022) and Herzig et al. (2024). The community was dominated by *Firmicutes* and *Desulfobacterota*, with *Bacteroidota, Proteobacteria*, and *Actinobacteriota* also present, of which common genera included *Desulfomicrobium, Clostridium sensu stricto 13, Proteiniphilum, Acetobacterium*, and *Pseudomonas* (Herzig et al., 2024). Minor taxa included *Firmicutes, Comamonadaceae, Clostridia*, and *Lactobacillales* (Fig. 1, Table 1).

For experiments, DD and LMC cultures were pre-incubated anaerobically for 7 days in broths A–D or P (Table 2) (Herzig et al., 2024), then transferred to fresh broth in sealed infusion bottles and supplemented with CH₄ before 14 days of anaerobic incubation. Salt concentrations in broths A–D were derived from previously described groundwater simulants: saline-reducing reference water (OL-SR, high salinity) and modified fresh water for reducing conditions (ALL-MR, low salinity/fresh water), based on measurements from the Finnish deep bedrock nuclear waste disposal site (Hellä et al., 2014). Broth P consisted of Postgate medium (PG), commonly used for SRB cultivation (Postgate, 1984). Broths differed in addition to salinity, in exogenous organic carbon source (ECS). Salt concentrations were 24 *g* L⁻¹ (OL-SR), 0.21 *g* L⁻¹ (ALL-MR), and 4.6 *g* L⁻¹ (PG) (Herzig et al., 2024). Triplicates and blanks (BAS) were included. After incubation, subsamples were analyzed for polar/semipolar metabolites (UHPLC-QTOFMS) and nutrient use was calculated by subtracting BAS metabolite concentrations. For UHPLC-QTOFMS, aliquots were filtered (0.2 μm), mixed with MeOH/H₂O and internal standards, evaporated, and reconstituted in MeOH:H₂O (70:30) (Herzig et al., 2024). Quality control samples were pooled with mean %RSD for identified metabolites of 22.1 %.

**Table 2:** Broths A-D and P used for the cultivation of DD and LMC.

Broth base salts (g/L):	OL-SR^1^	ALL-MR^1^	Postgate^2^		
CaCl_2_ · 2 H_2_O	15	0.019	0.10		
NaCl	12	0.056			
MgCl_2_ · 6 H_2_O	0.46	0.0060			
KCl	0.040	0.0075			
Na_2_SO_4_ · 10 H_2_O	0.012	0.028			
SrCl_2_ · 6 H_2_O	0.11				
H_3_BO_3_	0.0053				
NaF	0.0026				
NaBr	0.13				
KI	0.0011				
Na_2_SiO_3_ · 9 H_2_O		0.0080			
NaHCO_3_		0.090			
K_2_HPO_4_			0.50		
NH_4_Cl			1.0		
Na_2_SO_4_			1.0		
MgSO_4_ · 7 H_2_O			2.0		

Cultivation broths	Broth A	Broth B	Broth C	Broth D	Broth P
**Broth base:**	OL-SR	OL-SR	ALL-MR	ALL-MR	Postgate
**Broth additions:**					
Na-DL lactate, 2 g/L		x		x	x
Na-resazurin (0.1 % w/V), 0.5 mL/L	x	x	x	x	x
Na-thioglycolate, 0.1 g/L	x	x	x	x	x
Ascorbic acid, 0.1 g/L	x	x	x	x	x

1) Hellä et al., 2014, 2) Postgate, 1984.

### 2.2 Statistical analyses

Pearson correlation (R corrplot) (Wei and Simko, 2024) calculations at the *p* < 0.01 level were used to study the linear correlation between the bacterial communities and the utilized polar/semipolar metabolites. The Pearson correlation analyses were completed for the samples with *p* < 0.05 in the two-tailed T-test against baseline media (BAS) (see Supplement 1, Figure S1) using log transformed data of the percentage of used compound for data normalization. Only ASVs with sequence counts > 10 in the majority of the samples were considered in the calculations. Log transformed abundance percentage was used in all following calculations to normalize the sequence data. Further, log transformed data of polar/semipolar metabolites with *p* < 0.05 (two-tailed T-test) compared to BAS (%) were used to construct the metabolite fingerprint profiles of LMC and DD using the identified amino acids, other organic acids, phytohormones and tRNA constituents in the growth media in R using the pheatmap function (Kolde, 2018) and data from (Herzig et al., 2024). For these calculations, utilization was set as −1, production as +1 and for the samples for which either utilization or production at the *p* < 0.05 level in the two-tailed T-test against BAS was not observed, the value was set as zero. Orthogonal partial least squares discriminant analysis (OPLS‐DA) was carried out to investigate and visualize the pattern of metabolite changes between the ECS supplemented samples and the samples grown without ECS supplementation as well as the overall metabolism in the LMC compared to the metabolism in the reference strain DD. The OPLS‐DA model was evaluated by cross‐validation and for quality assessment the R2Y (goodness of fit of the model) and Q2 (predictive ability of the model) values were used and 2000-run permutations to test the possibility of obtaining those values for the goodness of fit and predictability by chance was used in MetaboAnalyst 6.0 (https://www.metaboanalyst.ca/) (Chong et al., 2018). In order to demonstrate the basic principles in LMC and DD metabolism adaptation log-transformed metabolite concentrations were analyzed in a hierarchical cluster analysis after grouping into two groups using Pearson correlation as similarity measure and Ward´s method for linkage analysis (Frimmersdorf et al., 2010; Brown et al., 2018) with top 12 features in MetaboAnalyst 6.0 using the Statistical Analysis module.

For addressing the metabolic pathways present we performed pathway and functional analyses using the Pathway and Functional Analysis modules of MetaboAnalyst 6.0 (Chong et al., 2019; Li et al., 2013). Log transformation was used for data normalization and *Psedomonas aeruginosa* PAO1 (KEGG) metabolome was selected as a reference pathway library. This was chosen based on the extensive metabolomic data available and as *Pseudomonas* ASVs were found throughout the LMC cultivations. Pathway impact values were obtained by analyzing the cumulative percentage upon matched metabolites. Accordingly, the top pathways were mapped into the Kyoto Encyclopedia of Genes and Genomes (KEGG) pathways.

In addition to identified metabolites, *m/z* values of all peaks were used in MetaboAnalyst 6.0 Functional analysis module. Similarly to the pathway analysis, log transformation was used for data normalization and *Pseudomonas aeruginosa* PAO1 as reference library.

## 3 Results

### 3.1 Metabolic fingerprinting

From the data 26 polar/semipolar compounds were identified, of which amounts were significantly different in samples DD and/or LMC compared with those in the BAS. Subsequently, eight of these were identified as amino acids (aspartic acid (Asp), glutamic acid (Glu), isoleucine (Ile), methionine (Met), proline (Pro), phenylalanine (Phe), tryptophan (Trp), valine (Val)), ten as other organic acids (2-hydroxybutyric acid (HBA), cis-dihomoaconitic acid (CAA), coumaric acid (CA), citric acid (CtA), fumaric acid (FA), hydroxyglutaric acid (Hyd), isovaleric acid (IVA), mevalonic acid (MVA), succinic acid (SA), itaconic acid ITA)), two as tRNA constituents (2-thiocytidine dihydrate (Thio), hypoxanthine (Hyp)), and three as phytohormones or their derivatives (5-hydroxyindole-3-acetic acid (5-HIAA), gibberellic acid (GA3), indole-3 acetic acid (IAA)). In addition, arbutin (ARB), catechin (Cat) and 3‑hydroxy-4–5-dimethylfuran-2(5H)-one (3HYD) were identified (Supplementary Table S1 and Figure S1).

**Fig. 2:**
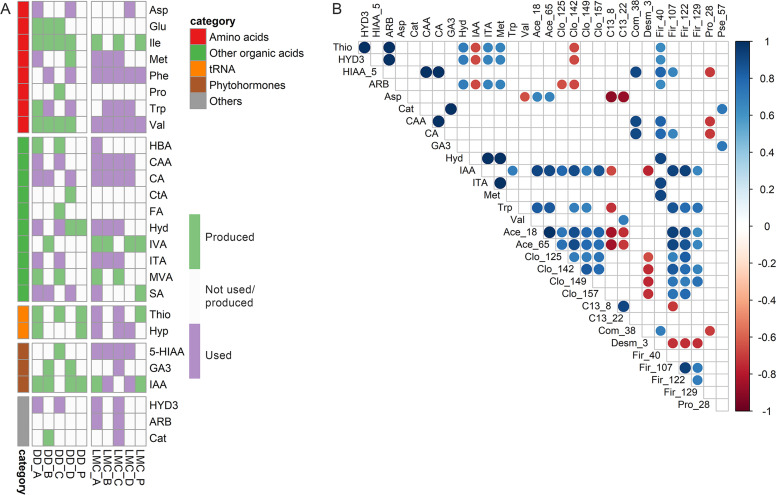
A) The metabolic fingerprint heatmap of DD and LMC based on polar/semipolar metabolite utilization/production. Heatmap was constructed from statistically significant (two-tailed *t*-test p-values < 0.05) metabolite utilization and production in the different broths. Profiles were constructed using previous data (Herzig et al., 2024, Supplement 3), including only metabolites with statistically significant utilization or production in at least one broth or enrichment culture. Metabolites not showing significant changes in any sample were assigned a value of zero in the profiles. For BAS polar/semipolar metabolite content, see Supplement 1 Figure S1. B) Correlation plots of identified polar metabolites utilized by LMC vs. bacterial communities. For correlation calculations log transformed data of the percentage of utilized metabolite present in BAS was used. The correlations significant at the *p* < 0.01 level are shown. For abbreviations, see Table 1. The numbers stand for a corresponding ASV.

Among the identified metabolites, seven were either utilized (*p* < 0.05) (Fig. 2A) in at least one of the samples in any of the set conditions (Asp, Phe, CAA, CA, ITA, 3HYD, ARB) or significant difference between BAS and any of the samples/conditions were not observed. Similarly, seven of the metabolites were deemed as produced (Glu, Ile, Pro, CtA, FA, IVA, MVA) and for 12 metabolites the production/utilization pattern was variable (i.e. in some samples/conditions they were utilized, while in others they were produced) (Met, Trp, Val, HBA, Hyd, SA, Thio, Hyp, 5-HIAA, GA3, IAA, Cat).

Metabolite utilization by the bacterial cultures depended on the applied culture conditions (broths A-D, P) and bacterial mixture (DD, LMC) (Fig. 2). Of the amino acids, Asp, Met, Phe and Trp were degraded by DD depending on the growth broth. Phe and Trp were utilized in ECS supplemented solutions B and D, while Met and Asp were utilized in broth A, lacking ECS supplement. In addition, Asp was utilized in broth D. Prominent other organic acid degradation was seen for DD especially in broth A, in which CAA, CA, Hyd, ITA and SA were utilized. CAA, Hyd and ITA were also utilized in broth C, and CA and SA in broths B and D. 3-hydroxy-4–5-dimetylfuran-2(5H)-one (HYD3) was utilized in broths A and C. Phytohormone and tRNA constituent utilization was absent in DD.

Unlike DD, LMC was featured by substantially increased organic acid, tRNA component and phytohormone utilization. Especially the overall amino acid metabolism in LMC was distinctive from DD with elevated utilization of Val, Met, Trp and Phe. Most notably Phe and Val were utilized in all broths by LMC and Met (A-C) and Trp (B-D) in the majority of the cultivation solutions. Similarly to DD, Asp was degraded in solution D. LMC also differed significantly from the reference strain DD in terms of the utilization of phytohormones and tRNA components. LMC degraded 5-HIAA in broths A-D, GA3 in broth C and IAA in broths B and D. In addition, tRNA constituents Thio and Hyp were utilized by LMC in broths A and C. Hyp was in addition utilized also in broth D.

In terms of the utilization of other organic acids, LMC and DD, however, shared some common features; corresponding utilization of CAA, CA, Hyd, ITA and SA was observed for both cultures. Though, LMC was able to degrade CAA, CA, Hyd and ITA in more varied conditions than DD. In addition, LMC degraded HYD3 and ARB in solutions A and C. From these HYD3 utilization in broths lacking the external carbon source (A and C) was the only systematically shared feature between LMC and DD. Cat was utilized by LMC in broth C.

Some positive correlations (*r* > 0.6, *p* < 0.01) between amino acids and bacteria, namely *Acetobacterium* (ASV_18 and 65), *Firmicutes* without further classification (ASV_40, 107, 122 and 129) and *Clostridium* sensu stricto 13 (ASV_22) were found (Fig. 2B). *Acetobacterium* (ASV_18 and 65) correlated positively with Asp utilization (*p* < 0.01), while *Firmicutes* ASV_40 had positive correlation with Met utilization. *Firmicutes* ASVs 107, 122 and 129 correlated positively with Trp utilization. *Clostridium* sensu stricto 13 (ASV_22) correlated positively with Val utilization.

Of the other organic acids, CAA, CA, Hyd and ITA utilization correlated positively with *Firmicutes* ASV_40, in addition CAA and CA had positive correlation with *Comamonadaceae* without further classification (ASV_38). CA also correlated with *Firmicutes* ASV_107. Further, 5-HIAA, HYD3, arbutin and thiocytidine utilization all correlated positively with ASV_40. *Firmicutes* ASV_107 and *Comamonadaceae* (ASV_38) had positive correlation with 5-HIAA. *Pseudomonas* (ASV_57) correlated with GA3 and Cat. However, most notably IAA utilization correlated positively with multiple bacterial ASVs; *Acetobacterium* (ASVs 18 and 65), *Clostridium* (ASVs 125, 142, 149 and 157), and *Firmicutes* (ASVs 107, 122 and 129).

### 3.2 Identifying the metabolic differences

The OPLS-DA model explained 81 % and 67 % of metabolite variability (R2X) in LMC and DD, respectively; In LMC first predictive component explained 93.6 % of the variance (R2Y) and was organized in two orthogonal components meanwhile 40.1 % of metabolite variation was predictive and aided in differentiating ECS supplemented cultivations from cultivations lacking ECS. In DD, 96.9 % of the variance was explained by the first predictive component, with one orthogonal component. Correspondingly 41.4 % of metabolite variation was predictive for DD. The model had a good overall fit with R2Y values of 0.998 and 0.994 and an overall cross-validation coefficient (Q2) of 0.994 and 0.989 for LMC and DD, respectively. The total metabolic profiles of polar/semipolar metabolites of LMC and DD under different growth conditions (ECS or NON) showed high variability between these two groups within 95 % confidence interval.

**Fig. 3:**
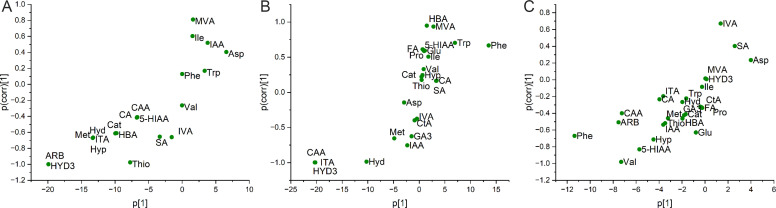
A) S-plots from Orthogonal PLS-DA (OPLS-DA) models showing significant metabolites contributing to group separation: (A) ECS vs. NON groups within the LMC community, (B) ECS vs. NON groups within the DD reference culture, and (C) overall discrimination between LMC and DD. Variables are plotted as covariance (p[1]) versus correlation (p(corr)[1]), highlighting metabolites with high VIP scores (>1) and significant contributions to group differentiation. *For abbreviations, see*Table 1*.*

On metabolite level LMC and DD shared some features for the significant metabolites separating the ECS and NON groups within the samples; HYD3, ITA and Hyd were among the most influential variables separating the groups for both samples (Fig. 3A and 3B). However, in LMC also arbutin (ARB), methionine (Met) and Hyp had high influence meanwhile in DD, in addition to HYD3, ITA an Hyd, also CAA had a high impact. Despite the shared features LMC clearly differed from the reference strain DD with 95 % confidence interval. The most significant metabolites defining the difference between the reference and LMC mix were the amino acids Phe and Val as well as ARB and CAA (Fig. 3C).

**Fig. 4:**
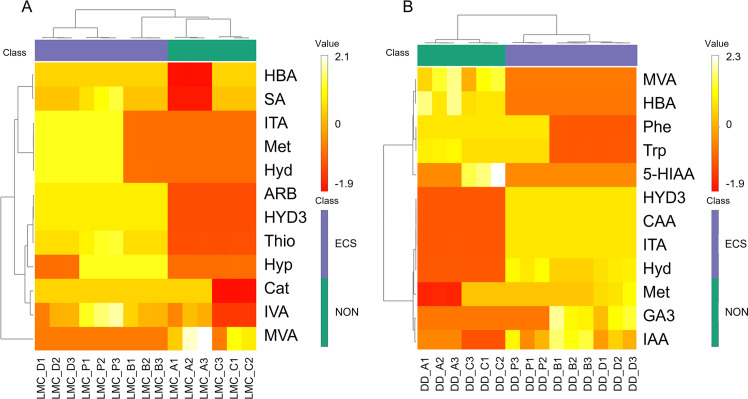
Unsupervised hierarchical clustering for treatment-driven metabolic differentiation assessment. Clustering was applied to log-transformed concentrations of the 12 most abundant polar and semipolar metabolites detected in differentially treated samples (A) LMC and (B) DD. Clustering employed Pearson correlation as the similarity measure and Ward’s method for linkage analysis. Metabolites were categorized into (i) those enriched in ECS-supplemented samples (purple) and (ii) those predominant in samples grown without ECS supplementation (green), revealing distinct metabolic signatures associated with ECS treatment and sample (LMC or DD) type. For abbreviations, see Table 1.

In regards of metabolic adaptations to specific growth conditions, two major cluster groups within the growth conditions were identified. The first group comprised of the samples lacking ESC, while the other main group represented the samples with ECS both in LMC and DD in accordance with the OPLS-DA. However, in LMC the ECS supplemented samples grown in high salt condition broth (broth B) also shared features with the samples grown in broths A and C lacking ECS supplement (Fig. 4A). In addition, two major clusters within the top 12 metabolic features were observed in DD, from which one group was characterized by amino acids Phe and Trp and organic acids MVA and HBA (Fig. 4B). The second group was more mixed, with organic acids CAA, ITA and Hyd combined with Met, HYD3 and phytohormones GA3 and IAA. The phytohormones though formed their own subcluster. LMC demonstrated two mixed major clusters, which were further divided into multiple subclusters (Fig. 4A). Most notably MVA formed its own major cluster.

### 3.3 Metabolite networks

**Fig. 5:**
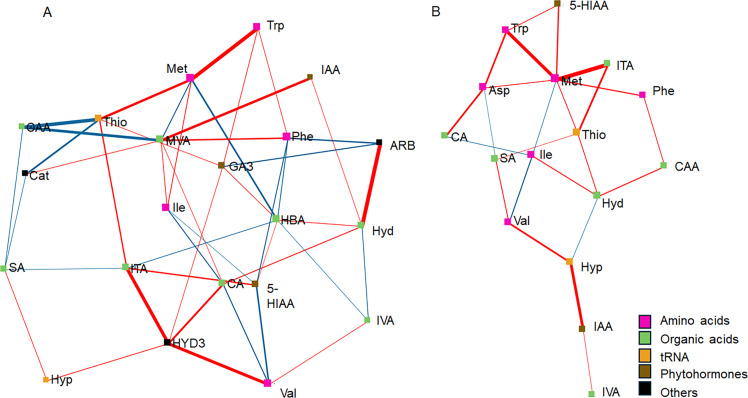
DSPC networks between produced and utilized metabolites in LMC without ECS (NON) (A) and with ECS (ECS) (B). In DSPC network analysis the nodes represent metabolites, edges represent the partial correlation of DSPC between two metabolites after conditioning on all other metabolites, and width of edges denote the partial correlation coefficient strengths (*p* < 0.05). Differential colors represent the classification of the metabolites. The red and blue lines indicate positive and negative correlations between two metabolites, respectively. For abbreviations, see Table 1.

**Fig. 6:**
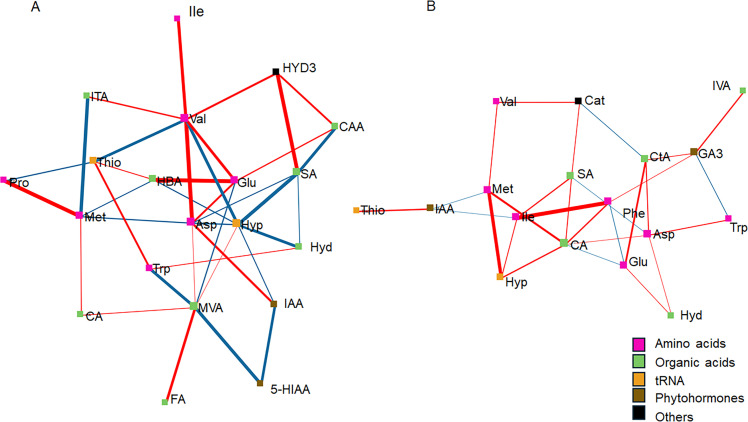
DSPC networks between produced and utilized metabolites in DD without ECS (NON) (A) and with ECS (ECS) (B). In DSPC network analysis the nodes represent metabolites, edges represent the partial correlation of DSPC between two metabolites after conditioning on all other metabolites, and width of edges denote the partial correlation coefficient strengths (*p* < 0.05). Differential colors represent the classification of the metabolites. The red and blue lines indicate positive and negative correlations between two metabolites, respectively. For abbreviations, see Table 1.

Dense interactions among amino acids, other organic acids, phytohormones and tRNA constituents occurred both in LMC (Fig. 5) and DD (Fig. 6). In LMC without ECS (Fig. 6A) MVA and HBA represented the main centers with high number of direct connections (seven) to other nodes (metabolites) in the network. However, only strong positive correlations (*p* < 0.01) were found between MVA and IAA. Other important nodes, with high degree of connections to other nodes were HYD3, ITA, GA3, CA, Thio, Phe, Hyd and Met, with each having five direct connections between other nodes within the network. Of these, HYD3 had a strong positive correlation with ITA and Val, Met with Trp, and Hyd with ARB. In ECS supplemented LMC networks the highest degree of connections was found for Met, with seven metabolites directly connected. Met had strong positive correlations with Val and ITA.

In DD without ECS supplement, MVA, Val, Asp and Hyp were the nodes having highest number of metabolites directly connected. Of these Val had strong positive correlations with HYD3, Glu, Ile, Asp and ITA (*p* < 0.01), while MVA had dense interactions with 5-HIAA and FA (*p* < 0.01). In addition, Asp correlated positively with IAA and Hyp with Hyd. In ECS treated DD samples the main hub was CA, with six metabolites directly connected, followed by Asp (5), Phe (4), GA3 (4), Ile (4), Met (4), CtA (4), SA (4), and Glu (4). Strong positive correlations were observed between Glu-CtA, CA-Met, Hyp-Met and Ile-Phe.

### 3.4 Metabolic pathways

**Fig. 7:**
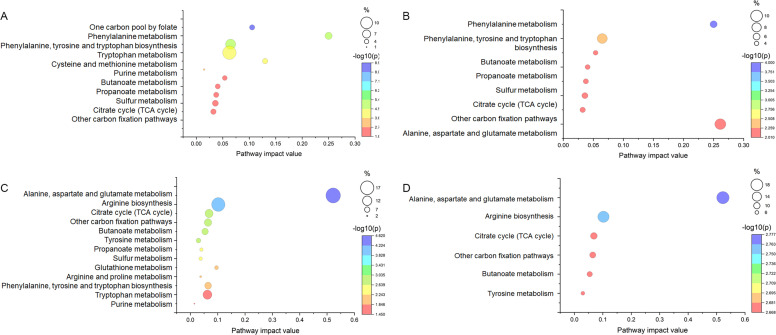
Pathway analysis in LMC cultivated without ECS supplement = NON (A), LMC cultivated with ECS supplement = ECS (B), DD cultivated without ECS supplement = NON (C) and DD cultivated with ECS supplement = ECS (D). Log transformation was used for data normalization and *Pseudomonas aeruginosa* PAO1 (KEGG) metabolome was selected as a reference pathway library. Pathway impact values were obtained by analyzing the cumulative percentage upon matched metabolites against BAS value. Only pathways with p-values < 0.01 are shown.

In LMC ECS supplemented (ECS) and cultivations without ECS (NON) the highest impact values were obtained for pathways linked to amino acid metabolism (Fig. 7A and B). Especially phenylalanine (NON and ECS samples) and alanine, aspartate and glutamate (in ECS samples) metabolism showed high impact values at around 0.25. In addition, sulfur metabolism and mixed carbon fixation pathways exhibited moderate to low impact (< 0.05) in both samples at *p* < 0.01 level. The most notably difference between LMC ECS and NON samples was that in ECS markedly lower number of significant metabolic pathways were present. Eight pathways had impact values > 0 in ECS. In the samples cultivated without ECS, the number of activated pathways increased to 11. Notably, cysteine and metabolism became more important in the ECS lacking cultivations.

DD showed some important differences from LMC; phenylalanine metabolism impact was low, meanwhile alanine, aspartate and glutamate metabolism showed high impact values (>0.5) in both DD cultivations (Fig. 7C and D). Unlike LMC, arginine metabolism had the second highest impact in both DD cultivations. However, its value remained moderate at around 0.1. In DD cultivations the impact of sulfur metabolism remained surprisingly low; under 0.05 in samples cultivated without ECS. In ECS supplemented samples the impact value of sulfur metabolism remained also under 0.05, but in addition it had p-value over 0.01.

Based on Holm-Bonferroni adjusted p-values (0.01) and impact values > 0.20, the most important pathways were phenylalanine metabolism in LMC and pathways linked to alanine, aspartate and glutamate metabolism in DD.

Further, in DD NON functional analysis showed high enrichment values for ascorbate, aldarate and butanoate metabolism (7.4 – 11.1, *p* < 0.05), while in all other samples functional analysis showed insignificant p-values (> 0.05) (Supplementary Table S2).

## 4 Discussion

The deep biosphere of the Fennoscandian Shield crystalline bedrock hosts extensive taxonomic diversity, and while genomic data increasingly reveal metabolic potential (e.g. Bell et al., 2022; Wu et al., 2016; Nyyssönen et al., 2014; Kietäväinen et al., 2025), few studies have examined metabolite utilization and production in deep terrestrial biosphere communities or enrichment cultures (Herzig et al., 2024; Bomberg et al., 2019). SRB are considered major contributors to energy flow in these ecosystems, yet overall metabolic dynamics remain poorly resolved. To address this gap, we investigated how environmental factors, such as salinity and carbon source composition, shape amino acid turnover, organic acid flux, and secondary metabolite profiles in enrichment cultures from the deep terrestrial subsurface (LMC culture). These community-level patterns, representing broader metabolic potential, were compared to those of a SRB reference strain, *Desulfovibrio desulfuricans* type strain 642 (DD culture).

### 4.1 Amino acid metabolic fingerprinting reveals response to changing environmental conditions in deep biosphere microbial communities

Both the LMC community and the DD pure culture exhibited similar growth-related metabolic shifts, with metabolite profiles primarily shaped by environmental conditions and the availability of extracellular carbon sources (ECS). Beyond this adaptive response, shared metabolic fingerprints were observed, notably the degradation of 3‑hydroxy-4,5-dimethylfuran-2(5H)-one (HYD3) and the production of mevalonate (MVA). Under nutrient-depleted conditions (broths A and C), both cultures transitioned toward HYD3 catabolism and MVA synthesis, suggesting a conserved adaptive strategy that reflects the metabolic versatility of these organisms.

The metabolic role of HYD3 remains uncertain and it is unclear whether furanic compounds are merely detoxified or actively metabolized as part of cellular energy pathways (Wierckx et al., 2011). In our network analysis, HYD3 showed strong interaction with valine (Val) both in LMC and DD. However, while in LMC Val was degraded in the reference SRB strain DD it was produced, meanwhile HYD3 was degraded in both the LMC population and in the reference strain. Any metabolic linkage between HYD3 and valine is not known, but furanones are known to suppress bacterial quorum sensing e.g. in *Pseudomonas aeruginosa* and *Chromobacterium violaceum* (Martinelli et al., 2004; Wu et al., 2004), both relevant in environmental bacterial communities, which in turn could affect amino acid metabolism (Slaughter, 1999).

Outside the similarities in HYD3 and mevalonate patterns, based on the OPSLS-DA model, the LMC community and the SRB reference strain DD were metabolically clearly distinct and in the hierarchical clustering model these cultivations formed clearly distinct metabolite clusters, characterized by different amino acids; both LMC and DD utilized metabolic pathways related to amino acids, but relative impact of distinct amino acid pathways differed between the bacterial mix and the *D. desulfovibrio* strain.

### 4.2 High‑impact metabolic pathways define the deep terrestrial subsurface community

Amino acids are essential for bacterial growth and in general bacteria can utilize a variety of amino acids, but the utilization patterns differ substantially depending on the bacterium or strain in question, and are influenced by in addition to growth conditions, also by the growth phase (Muranaka et al., 2023; Bonnet et al., 2020).

In our data the reference strain DD utilized aspartate (Asp) in broths A and D, and for glutamate (Glu) only biosynthesis was observed. In the LMC population, the utilization of phenylalanine (Phe) and valine (Val) was substantially increased, and this trend was consistent across all cultivation conditions. Asp in general, is a non-essential amino acid, but is utilized e.g. as a nitrogen and energy source by various bacteria (Oikawa, 2006). Glu, however, is important for many bacterial metabolic activities and in particular in responses to environmental stress (Feehily and Karatzas, 2013). The elevated Glu biosynthesis observed in DD, particularly under conditions deviating from the standard Postgate medium for *Desulfovibrio*, likely reflects an adaptive response to stress imposed by the changes in the experimental environment.

The further pathway analysis displayed alanine (Ala), Asp and Glu metabolism with high impact in the SRB reference DD. In LMC, Phe metabolism showed the highest pathway impact, and cysteine (Cys) and methionine (Met) metabolism became important especially in samples without ECS supplement.

Pathways with high impact values are central to the metabolic changes (Wieder et al., 2021), which was observed in the LMC community. However, Wieder et al. (2021) show that the analysis is influenced by the choice of reference organism and pathway set, which is complicated by the diverse bacterial composition of LMC. Using the *Pseudomonas aeruginosa* metabolome as a reference provides only a preliminary model, but these high-impact pathways likely play key roles in the overall metabolic network of deep subsurface bacterial populations. In addition, the high impact values suggest these pathways could serve as biomarkers for assessing and monitoring the community’s metabolic state. Moreover, significant differences between pathways in LMC and those in the reference SRB DD indicate that this approach may also help distinguish between different community types.

In general, the pathway analysis of LMC and DD in our study indicates that Ala–Asp–Glu metabolism constitutes a core pathway in the SRB strain DD under the nutrient conditions tested, whereas Phe metabolism, together with Val utilization, appears central to the LMC community, which we expect to more closely reflect the metabolic patterns typical of the deep terrestrial subsurface (DTS).

### 4.3 Distinct amino acid catabolism and biomineralisation pathways define deep subsurface community metabolism

During anaerobic respiration amino acids are broken down based on the existing metabolic pathways (Schwendner et al., 2022). The degradation rate is influenced not only by the accessibility of specific amino acids but also by the presence of other metabolizable organic compounds, such as lactate. Therefore, amino acid degradation has been proposed as a potential biosignature, as microorganisms preferentially catabolize specific amino acids available in their environment (Schwendner et al., 2022). Although the amino acid biosignature might overlap with the amino acids synthesized by the organism itself, it still indicates the degradation of the amino acid pool surrounding the specific bacterium or community (Schwender et al., 2022). Additionally, a change in amino acid concentrations from the expected background abiotic levels (such as the BAS concentration) serves as an indicative signature of metabolic processes (Schwendner et al., 2022).

In general, a substantial difference in amino acid catabolism was observed when comparing LMC to the DD pure culture. DD degraded Phe and Trp only in ECS supplemented solutions (B and D), and Asp and Met in the high salinity solution A. Yet, the more notable characteristic was the production of Glu, isoleucine (Ile), and Val under most cultivation conditions in DD. This was substantially different from LMC population, in which amino acids were mostly degraded. In LMC the amino acid catabolism was most pronounced for Phe and Val, but in addition Met and Trp were degraded under most of the studied conditions.

The metabolic fate of the amino acids remains unknown, but besides being incorporated into bacterial biomass, dissolved free amino acids can also be mineralized into ammonium (NH_4_^+^) and CO_2_ (Hansen and Blackburn, 1995; Hassan et al., 2025). Although our data lacks information on NH_4_^+^, the DD pure culture showed increased CO_2_ production in solutions containing ECS (corresponding to solutions with increased Phe and Trp degradation). A similar trend was observed for the LMC. In general, during amino acid ammonification, microbial metabolism produces CO_2_ and ammonia (NH_3_), which after NH_3_ hydrolyzes into NH_4_^+^ and hydroxide (OH^-^) ions, promoting precipitation of carbonate biominerals such as calcite (CaCO₃), a process known to occur in SRB (Gai et al., 2014; Paul et al., 2017). Biomineralization was indeed observed in DD SRB pure culture (see Supplementary analysis), although whether the produced CO_2_ participates in the formation of these precipitates as well as the characteristics of the observed crystals are yet to be determined.

#### 4.3.1 Degradation of aromatic amino acids

Generally, aromatic amino acids such as phenylalanine and tryptophan are used as substrates by various microorganisms (Schneider et al., 1997), and the oxidation of these amino acids involving oxygenase enzymes under oxic conditions is well-documented (e.g. Gottschalk, 1985). However, under anoxic conditions, the aromatic ring poses an additional challenge for bacterial degradation, as its stabilizing resonance energy requires specialized strategies to overcome (Teufel et al., 2010). In our study, aromatic amino acid degradation was markedly higher in the multi-genera LMC community than in the DD pure culture, suggesting that taxa other than SRB drive this process. This interpretation is supported by the positive correlation between Trp degradation and *Firmicutes* ASVs (107, 122, 129), and aligns with existing knowledge, as aromatic amino acid catabolism has not been reported for SRB. Therefore, the observed degradation of aromatic amino acids by the multi-genera LMC community suggests that such catabolic activity may be more prevalent in deep terrestrial subsurface environments than previously assumed, given the traditional view of SRB-dominated metabolism in these ecosystems.

#### 4.3.2 Cysteine and methionine metabolism in connection with sulfur metabolism

Methionine (Met) was extensively degraded by LMC population in solutions A - C, which was markedly different from the metabolism observed in the reference strain DD. In addition, cysteine (Cys) – Met metabolism was among the most important metabolic pathways, along with the sulfur metabolism, in the LMC cultivated without the ECS supplement. While Met was degraded by LMC, lower percentage of available free sulfate was utilized (see Herzig et al., 2024) compared to the other nutrient broths (D and P), in which Met degradation was not observed.

In general, Met – Cys and sulfur (S) metabolism are intertwined and in the environmental bacterial communities, the Met – Cys – S metabolism is characterized by mutualistic relationships (e.g. Stipanuk and Ueki, 2011; Kohlmeier, 2015). These relationships depend on complex molecular interactions, where metabolites produced by one microbe might influence the entire community. In LMC, a positive correlation between Met degradation and *Firmicutes* ASV_40 was observed, suggesting a potential role in sulfur metabolism within this community.

Beyond sulfur metabolism, cysteine and methionine pathways are connected to isoprenoid biosynthesis via 1-deoxy-d-xylulose 5-phosphate in the DOXP/MEP pathway (Kanehisa et al., 2025 /KEGG map 00,900 + M00364), a route characteristic of prokaryotes (Orsi et al., 2020). Earlier we discussed the significance of mevalonate (MVA) and MEP pathways as potential stress regulators in these communities, as MVA accumulation was observed in the growth solutions lacking ECS (Herzig et al., 2024). Increased Met catabolism observed in this study may indicate activation of the MEP pathway alongside the MVA and overall sulfur cycle. Co-expression of both isoprenoid pathways have been reported e.g. in *Rhodobacter sphaeroides* and the cyanobacterium *Synechocystis* (Bentley et al., 2014a; Bentley et al., 2014b; Orsi et al., 2020). Given that *Rhodobacter* species were also detected in the LMC community, the simultaneous occurrence of these pathways could, at least partly, be connected to the Cys - Met metabolism highlighted in the current pathway analysis. These pathways produce key intermediates like isopentenyl pyrophosphate (IPP) and dimethylallyl pyrophosphate (DMAPP), which serve as essential building blocks for isoprenoids (Holstein et al., 2004).

## 5 Conclusions

Although DTS enrichment cultures do not fully replicate in situ communities, they provide a closer approximation than reference strains alone and, when combined with these strains, allow assessment of metabolic shifts across the broader community. Our metabolic fingerprinting revealed clear distinctions between the DTS enrichment culture and the reference strain, as well as individual bacterial taxa and growth conditions. The DTS community exhibited distinct metabolic features compared to *Desulfovibrio desulfuricans* type strain 642, with core metabolic processes centered around phenylalanine, cysteine, and methionine metabolism. Extensive amino acid and other organic acid degradation was observed, with phenylalanine and valine degradation constituting the core metabolic processes, largely unaffected by nutrient changes. In contrast, the metabolism of 3‑hydroxy-4,5-dimethylfuran-2(5H)-one, arbutin, mevalonate, thiocytidine, and isoleucine was strongly influenced by external organic carbon availability, indicating the overall importance of these pathways in the metabolic regulation in the wider DTS communities.

## Credit author statement

Merja Herzig: Conceptualization; writing – original draft; methodology; validation; visualization; writing – review and editing; software; formal analysis; data curation; investigation. Tuulia Hyötyläinen: Investigation; writing – review and editing; methodology. Malin Bomberg: Conceptualization; investigation; writing – review and editing; methodology.

## Declaration of competing interest

The authors declare that they have no known competing financial interests or personal relationships that could have appeared to influence the work reported in this paper.

## Appendix Supplementary materials

Image, application 1
