# Supplementary material for: Microbial metabolism in deep terrestrial subsurface communities - amino acids as biosignatures
Source: Curr Res Microb Sci. 2026 Jan 3;10:100547. doi: 10.1016/j.crmicr.2026.100547 (PMC12811484; doi:10.1016/j.crmicr.2026.100547)
Supplement: Supplementary file 1 [file mmc1.docx]

**Supplementary analysis**

**Carbonate precipitation/Supplementary Biomineralization studies**

In order to study the carbonate biomineralization potential of SRB, *D. desulfuricans* (type strain 642) (DD) was cultivated in Postgate medium (broth P) (Herzig et al. 2024) amendmed with 41 µM of NaHCO_3_ for 21 days. After incubation, the samples were prepared for transmission electron microscopy (TEM) as previously described in Lusa et al. (2017).

After incubation, crystalline precipitations were observed in these samples (Figure SA1). No corresponding crystals were found in the control samples incubated without bacterial amendments.


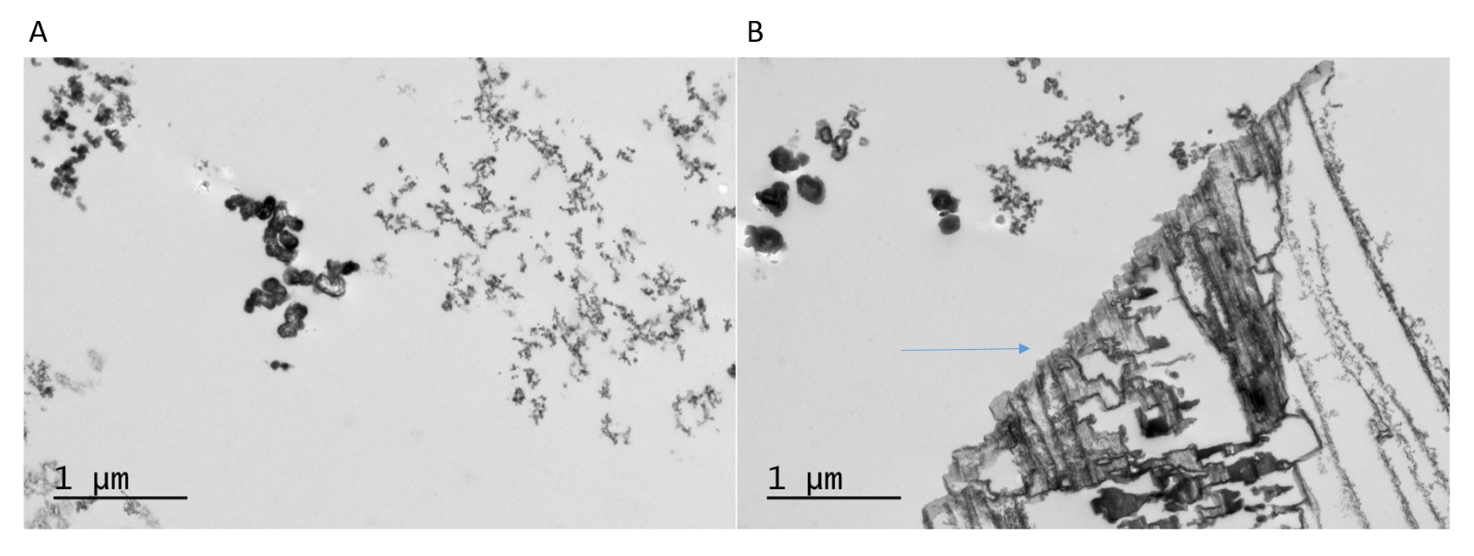


**Figure SA1.** *A) Sample incubated without SRB (crtl), B) Crystalline precipitations in SRB containing samples after incubation of 21 days in carbonate amended Postgate medium.*

**Supplement**

**
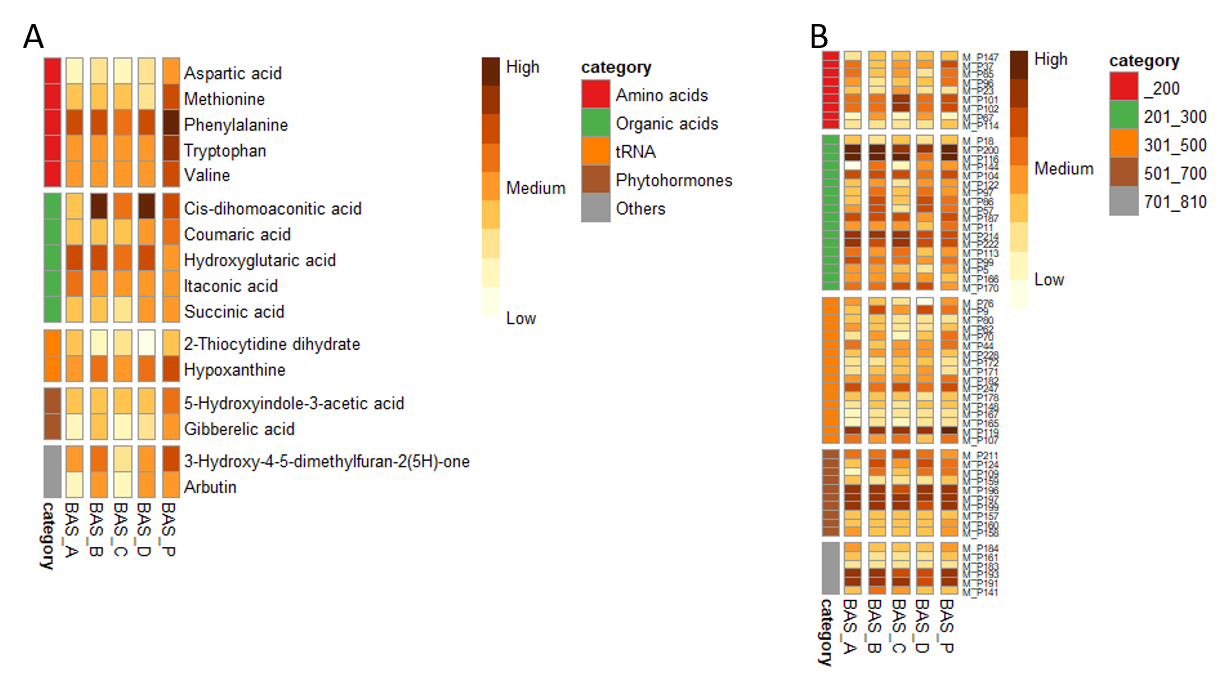
Figure S1.** *Identified polar/semipolar metabolites in BAS (A). The polar/semipolar metabolites without further identification are presented in the order of increasing molecular weight (m/z) (B). Only the detected metabolites that were used by the bacteria at the p<0.01 level are shown in the diagram.*

**Table S1*.*** *Identified polar/semipolar metabolites in growth solution A – D and P in DD and LMC cultures that were used to construct the metabolic fingerprints of DD and LMC.*

| **m/z** | **retention time** | **main ID** | **Short** |
| --- | --- | --- | --- |
| 103.0401 | 1.00 | 2-Hydroxybutyric acid | 2-HBA |
| 258.0843 | 2.00 | 2-Thiocytidine dihydrate | Thio |
| 206.9914 | 3.00 | 3-Hydroxy-4-5-dimethylfuran-2(5H)-one | 3HYD |
| 151.041 | 5.00 | 4-Hydroxyphenylacetic acid | 4-HPAA |
| 190.0446 | 6.00 | 5-Hydroxyindole-3-acetic acid | 5-HIAA |
| 271.0786 | 7.00 | Arbutin | ARB |
| 132.0306 | 8.00 | Aspartic acid | Asp |
| 289.0791 | 9.00 | Catechin | CAT |
| 201.0358 | 10.00 | cis-dihomoaconitic acid | CAA |
| 191.0272 | 11.00 | Citric acid | CtA |
| 163.0405 | 12.00 | Coumaric acid | CA |
| 115.0399 | 14.00 | Fumaric acid | FA |
| 345.1417 | 15.00 | Gibberelic acid | GA3 |
| 146.0462 | 16.00 | Glutamic acid | Glu |
| 147.0302 | 17.00 | Hydroxyglutaric acid | Hyd |
| 135.0301 | 19.00 | Hypoxanthine | Hyp |
| 174.0571 | 22.00 | Indole-3-acetic acid | IAA |
| 130.0646 | 24.00 | Isoleucine | Ile |
| 101.0609 | 25.00 | Isovaleric acid | IVA |
| 129.0195 | 26.00 | Itaconic acid | ITA |
| 148.0434 | 27.00 | Methionine | Met |
| 147.0659 | 28.00 | Mevalonic acid | MVA |
| 164.0717 | 29.00 | Phenylalanine | Phe |
| 114.0562 | 30.00 | Proline | Pro |
| 117.0198 | 31.00 | Succinic acid | SA |
| 203.0831 | 32.00 | Tryptophan | Trp |
| 116.0717 | 33.00 | Valine | Val |

**Table S2.** *Functional analysis in LMC cultivated without ECS supplement = NON, LMC cultivated with ECS supplement = ECS, DD cultivated without ECS supplement = NON and DD cultivated with ECS supplement = ECS. Log transformation was used for data normalization and Pseudomonas aeruginosa PA01 (KEGG) metabolome was selected as a reference pathway library.*

| **LMC NON** |  |  | **LMC ECS** |  |  |
| --- | --- | --- | --- | --- | --- |
|  | Enrichment | p-value |  | Enrichment | p-value |
| C5-Branched dibasic acid metabolism | 10.4 | 0.235 | Galactose metabolism | 4.8 | 0.387 |
| Ascorbate and aldarate metabolism | 7.8 | 0.129 | Glycerolipid metabolism | 4.8 | 0.627 |
| Carbapenem biosynthesis | 6.9 | 0.763 | Phenylalanine metabolism | 4.3 | 0.161 |
| Biosynthesis of various plant secondary metabolites | 6.9 | 0.258 | Biosynthesis of various plant secondary metabolites | 4.0 | 0.627 |
| Valine, leucine and isoleucine biosynthesis | 6.6 | 0.176 | Phenylalanine, tyrosine and tryptophan biosynthesis | 3.0 | 0.161 |
| Naphthalene degradation | 5.2 | 0.946 | Ascorbate and aldarate metabolism | 3.0 | 0.774 |
| Butanoate metabolism | 5.2 | 0.062 | Inositol phosphate metabolism | 3.0 | 0.387 |
| D-Amino acid metabolism | 4.2 | 0.235 | Toluene degradation | 3.0 | 0.864 |
| Pantothenate and CoA biosynthesis | 2.8 | 0.681 | D-Amino acid metabolism | 2.9 | 0.556 |
| Lysine degradation | 2.8 | 0.832 | Streptomycin biosynthesis | 2.7 | 0.627 |
| Toluene degradation | 2.6 | 0.510 | Xylene degradation | 2.4 | 0.627 |
| Phenylalanine, tyrosine and tryptophan biosynthesis | 2.6 | 0.324 | C5-Branched dibasic acid metabolism | 2.4 | 0.971 |
| Phenylalanine metabolism | 2.4 | 0.707 | Pentose and glucuronate interconversions | 2.2 | 0.627 |
| Tyrosine metabolism | 2.4 | 0.665 | Starch and sucrose metabolism | 2.2 | 0.627 |
| Taurine and hypotaurine metabolism | 2.3 | 0.510 | Styrene degradation | 2.2 | 0.627 |
| Xylene degradation | 2.1 | 0.763 | Valine, leucine and isoleucine biosynthesis | 2.2 | 0.953 |
| Alanine, aspartate and glutamate metabolism | 1.9 | 0.707 | Tyrosine metabolism | 2.1 | 0.665 |
| Sulfur metabolism | 1.9 | 0.258 | Butanoate metabolism | 2.0 | 0.889 |
| Pentose and glucuronate interconversions | 1.9 | 0.763 | Lysine degradation | 1.6 | 0.919 |
| Styrene degradation | 1.9 | 0.763 | Valine, leucine and isoleucine degradation | 1.6 | 0.833 |
| One carbon pool by folate | 1.8 | 0.258 | Fructose and mannose metabolism | 1.5 | 0.774 |
| Monobactam biosynthesis | 1.7 | 0.946 | Aminobenzoate degradation | 1.3 | 0.627 |
| Arginine and proline metabolism | 1.6 | 0.930 | Chlorocyclohexane and chlorobenzene degradation | 1.3 | 0.387 |
| Nitrogen metabolism | 1.5 | 0.510 | Amino sugar and nucleotide sugar metabolism | 1.2 | 0.504 |
| Glyoxylate and dicarboxylate metabolism | 1.4 | 0.515 | Glycolysis or Gluconeogenesis | 1.1 | 0.627 |
| Lysine biosynthesis | 1.4 | 0.886 | Pyruvate metabolism | 1.1 | 0.774 |
| Glutathione metabolism | 1.4 | 0.510 | Propanoate metabolism | 1.1 | 0.864 |
| Valine, leucine and isoleucine degradation | 1.3 | 0.907 | Pantothenate and CoA biosynthesis | 1.1 | 0.952 |
| Citrate cycle (TCA cycle) | 1.3 | 0.510 | One carbon pool by folate | 1.0 | 0.627 |
| Nicotinate and nicotinamide metabolism | 1.3 | 0.886 | O-Antigen nucleotide sugar biosynthesis | 0.9 | 0.387 |
| Glycine, serine and threonine metabolism | 1.3 | 0.907 | Pentose phosphate pathway | 0.8 | 0.864 |
| Arginine biosynthesis | 1.2 | 0.886 | Glycine, serine and threonine metabolism | 0.7 | 0.952 |
| Chlorocyclohexane and chlorobenzene degradation | 1.1 | 0.510 | Purine metabolism | 0.3 | 0.864 |
| Histidine metabolism | 1.0 | 0.510 |  |  |  |
| Propanoate metabolism | 1.0 | 0.946 |  |  |  |
| Other carbon fixation pathways | 0.9 | 0.510 |  |  |  |
| Cysteine and methionine metabolism | 0.9 | 0.832 |  |  |  |
| Porphyrin metabolism | 0.3 | 0.886 |  |  |  |
|  |  |  |  |  |  |
| **DD NON** |  |  | **DD ECS** |  |  |
|  | Enrichment | p-value |  | Enrichment | p-value |
| C5-Branched dibasic acid metabolism | 11.9 | 0.198 | Biosynthesis of various plant secondary metabolites | 8.6 | 0.337 |
| **Ascorbate and aldarate metabolism** | **11.1** | **0.042** | Ascorbate and aldarate metabolism | 6.5 | 0.462 |
| Valine, leucine and isoleucine biosynthesis | 8.1 | 0.088 | Phenylalanine metabolism | 6.1 | 0.155 |
| **Butanoate metabolism** | **7.4** | **0.022** | Arginine biosynthesis | 5.8 | 0.087 |
| Pantothenate and CoA biosynthesis | 5.4 | 0.114 | C5-Branched dibasic acid metabolism | 5.2 | 0.772 |
| Monobactam biosynthesis | 4.9 | 0.444 | Pantothenate and CoA biosynthesis | 4.7 | 0.307 |
| Lysine biosynthesis | 4.0 | 0.285 | Styrene degradation | 4.7 | 0.337 |
| Lysine degradation | 4.0 | 0.582 | Monobactam biosynthesis | 4.3 | 0.565 |
| Nicotinate and nicotinamide metabolism | 3.7 | 0.285 | Lysine biosynthesis | 3.5 | 0.462 |
| Valine, leucine and isoleucine degradation | 2.9 | 0.477 | Lysine degradation | 3.5 | 0.649 |
| Glycine, serine and threonine metabolism | 2.7 | 0.358 | beta-Alanine metabolism | 3.5 | 0.185 |
| Alanine, aspartate and glutamate metabolism | 2.7 | 0.444 | Nicotinate and nicotinamide metabolism | 3.2 | 0.462 |
| Pentose and glucuronate interconversions | 2.7 | 0.584 | Cyanoamino acid metabolism | 2.9 | 0.337 |
| Styrene degradation | 2.7 | 0.584 | Aminobenzoate degradation | 2.9 | 0.337 |
| beta-Alanine metabolism | 2.0 | 0.354 | Alanine, aspartate and glutamate metabolism | 2.4 | 0.565 |
| Citrate cycle (TCA cycle) | 1.9 | 0.354 | Valine, leucine and isoleucine biosynthesis | 2.4 | 0.883 |
| Arginine biosynthesis | 1.6 | 0.734 | Phenylalanine, tyrosine and tryptophan biosynthesis | 2.2 | 0.565 |
| Cyanoamino acid metabolism | 1.6 | 0.584 | Butanoate metabolism | 2.2 | 0.817 |
| Chlorocyclohexane and chlorobenzene degradation | 1.6 | 0.354 | D-Amino acid metabolism | 2.1 | 0.772 |
| Propanoate metabolism | 1.4 | 0.831 | Glycine, serine and threonine metabolism | 1.6 | 0.717 |
| Cysteine and methionine metabolism | 1.3 | 0.582 | Tyrosine metabolism | 1.5 | 0.817 |
| Other carbon fixation pathways | 1.3 | 0.354 | Arginine and proline metabolism | 1.3 | 0.854 |
| Sulfur metabolism | 1.3 | 0.584 | Cysteine and methionine metabolism | 1.2 | 0.649 |
| One carbon pool by folate | 1.3 | 0.584 |  |  |  |
| D-Amino acid metabolism | 1.2 | 0.958 |  |  |  |
| Glyoxylate and dicarboxylate metabolism | 1.0 | 0.734 |  |  |  |
| Tyrosine metabolism | 0.8 | 0.974 |  |  |  |
| Arginine and proline metabolism | 0.8 | 0.984 |  |  |  |
